# A new data integration framework for Covid-19 social media information

**DOI:** 10.1038/s41598-023-33141-y

**Published:** 2023-04-15

**Authors:** Lauren Ansell, Luciana Dalla Valle

**Affiliations:** School of Engineering, Computing and Mathematics, University of Plymouth, Plymouth, PL48AA UK

**Keywords:** Mathematics and computing, Statistics, Viral infection

## Abstract

The Covid-19 pandemic presents a serious threat to people’s health, resulting in over 250 million confirmed cases and over 5 million deaths globally. To reduce the burden on national health care systems and to mitigate the effects of the outbreak, accurate modelling and forecasting methods for short- and long-term health demand are needed to inform government interventions aiming at curbing the pandemic. Current research on Covid-19 is typically based on a single source of information, specifically on structured historical pandemic data. Other studies are exclusively focused on unstructured online retrieved insights, such as data available from social media. However, the combined use of structured and unstructured information is still uncharted. This paper aims at filling this gap, by leveraging historical and social media information with a novel data integration methodology. The proposed approach is based on vine copulas, which allow us to exploit the dependencies between different sources of information. We apply the methodology to combine structured datasets retrieved from official sources and a big unstructured dataset of information collected from social media. The results show that the combined use of official and online generated information contributes to yield a more accurate assessment of the evolution of the Covid-19 pandemic, compared to the sole use of official data.

## Introduction

The outbreak of the Covid-19 disease infected and killed millions of people globally, resulting in a pandemic with enormous global impact. This disease affects the respiratory system, and the viral agent that causes it spreads through droplets of saliva, as well as through coughing and sneezing. As an extremely transmissible viral infection, Covid-19 is causing significant damage to countries’ economies because of its direct impact on the health of citizens and the containment measures taken to curtail the virus. In the UK, Covid-19 had serious implications for people’s health and the healthcare services, with more than 8 million confirmed cases and 150,000 deaths, as government figures show. There is thus a widespread interest in accurately estimating and assessing the evolution of the pandemic over time.

Current studies on Covid-19 are typically based on a single source of information. Most of them implemented quantitative analyses focusing on historical data to produce forecasts of the pandemic. For example, clinical data of Covid-19 patients was used to statistically analyse with meta-analysis the clinical symptoms and laboratory results aiming at explaining the discharge and fatality rate^1^. Another study considered data on confirmed and recovered cases and deaths, the growth rate and the trend of the disease in Australia, Italy and the UK^2^. The authors predicted epidemiology in the three countries with mathematical approaches based on susceptible, infected, and recovered (SIR) cases and susceptible, exposed, infected, quarantined, and recovered (SEIQR) cases, comparing them with the Prophet machine learning algorithm and the logistic regression. Machine learning methods were also adopted to implement logistic regression and gradient boosted trees on health risk assessment questionnaires and medical claims diagnosis data to predict complications due to Covid-19^3^.

However, some authors in the literature criticize approaches which are exclusively based on official quantitative information. Existing Covid-19 studies based on historical data and prediction models for the pandemic are “poorly reported, at high risk of bias and underperforming”^4,5^.

Text data in the form of social media insights are used in the literature to evaluate and predict the progression of the Covid-19 pandemic. For example, a lag correlation analysis was employed with data collected from Google Trends, Baidu Search Index and Sina Weibo Index^6^. Sina Weibo messages were also analysed in other studies^7–9^. A statistical analysis based on the Fisher exact test was carried out, the rates of death were calculated with the Kaplan–Meier method and risk factors for mortality were established using the multivariate Cox regression^7^. A kernel density analysis and an ordinary least square regression were implemented to identify the spatiotemporal distribution of Covid-19 cases in the main urban area of Wuhan, China^8^. Descriptive statistics were calculated and a time series analysis was applied to the data^9^. Baidu Search Index information was analysed using different statistical methods, including the subset selection, forward selection, lasso regression, ridge regression and elastic net^10^. Google trends searches, Wikipedia page views and Twitter messages were gathered implementing a regression analysis to show that online-retrieved information provided a leading indication of the number of people in the USA who became infected and die from the coronavirus^11^.

However, contributions in the literature which are exclusively based on either official or online information as stand-alone sources are not taking into account the drawbacks affecting the data and the potential synergies between different data sources. On the one hand, due to limited capacity of testing, official data on confirmed cases are unlikely to reflect the true Covid-19 numbers. On the other hand, social media data are generated by users on a voluntary basis and may not capture information about the entire population. Therefore, predictive models built on a single source of information might generate biased results.

The goal of this paper is to develop a state-of-the-art data integration framework, leveraging the dependencies between historical and online data to provide more accurate evaluations of the Covid-19 dynamics. The proposed approach exploits the synergies between official and online generated Covid-19-related data, yielding improved predictions of the pandemic compared to forecasts obtained solely with a single source of information.

Our approach is based on vine copulas, which are very flexible mathematical tools, able to correctly capture the dependence structures between different variables^12^. Integration of different data sources using copulas and Bayesian networks was proposed in the literature^13–15^. However, the approach adopted by the authors was based on data calibration^16^. In this paper, our aim is to propose a comprehensive novel data integration framework, able to improve data modelling and forecasting^17^.

So far, the application of copulas and vines to pandemic data has been limited to study the implications of Covid-19, especially in the financial field, rather than to directly calculate forecasts of pandemic trends. For example, a Markov-switching dynamic copula with Student-t distribution was implemented to explore Covid-19 shock effects on energy markets^18^. A quantile regression model estimated via vine copula was used to show that speculation in energy and precious metal futures are more prevalent in crisis periods such as the Covid-19 pandemic^19^.

The vine copula approach allows us to obtain the joint multivariate distribution of the marginals, embedding the actual dependence structure between them. The marginals can belong to any distributional family and can potentially be all different. Vine copulas use bivariate (pair) copulas as building blocks. Since there exist a wide variety of bivariate copula families, able to capture as symmetric as well as asymmetric dependencies, the vine copula approach is highly flexible, allowing us to model virtually any type of dependence structures between each pair of variables^12^.

This paper proposes a novel data integration methodology based on vine copulas, able to exploit the synergies between two different types of information: (a) official and (b) social media data. As official information (a) we consider structured historical pandemic data and, as social media data (b), we consider a large unstructured online-retrieved dataset on Covid-19, relevant to the UK geographical area. We compare three models: (i) the flexible vine copula approach, where all pair copulas are different and are able to capture any type of dependencies between variables; (ii) the Gaussian copula approach, where all pair copulas are set to be equal to the Gaussian copula, and hence are only able to capture symmetric dependence; (iii) the independence approach, where we assume absence of dependence between the variables (in particular, no dependence between the official and social media data). Models (ii) and (iii) are traditional approaches, which impose restrictions on the relationships between variables. In particular, the Gaussian model (ii) assumes the existence of relations between official (a) and social media data (b), yet it allows only the traditional Gaussian family of distributions to describe this relationship. The independence model (iii), instead, does not allow the existence of any form of dependence between the variables and, in particular, between those derived by official (a) and social media (b). We are particularly interested in assessing the contribution of online-generated data (b) to the evaluation and prediction of the Covid-19 pandemic, hence in the performance of models (i) and (ii) compared to model (iii). Vine copulas allow us to compute predictions based on the joint multivariate distribution of the marginals. Differently from regression models, all marginals in vine copula models hold the same role and can be used in turn to predict selected variables. Here, we are specifically interested to evaluate how social media information (b) perform in predicting official pandemic data (a). Our results will show that the vine copula approaches (i) and (ii) perform better than the independence traditional approach (iii), which does not take into account associations between official and on-line information to estimate and predict the Covid-19 dynamic.

## Dataset

The structured and unstructured data used in this paper were collected daily between the 21st April 2020 and the 9th May 2021. As structured official data (a), we considered the following 6 variables: the number of new admitted patients (Admissions), the number of hospital cases (Hospital), the number of patients on ventilation (ICU_Beds), the number of tests (VirusTests), the number of positive cases (Cases) and the number of deaths (Deaths). The first four data variables were gathered from the UK Government dashboard, while the last two variables were downloaded from the Johns Hopkins University database. This information was available in cumulative form, therefore to obtain the daily time series, the previous days total was subtracted from the current days total. As unstructured online data (b), we collected Google Trends information on the number of searches for the keywords *Covid-19*, *coronavirus*, *first wave*, *second wave* and *variant*, using the gtrendsR package from the R software^20,21^. In particular, the function gtrends from the R package allows us to specify the Google Trends query keywords, the geographical region (in this case “GB”) and the timespan of the query. In addition, we retrieved Twitter messages containing the same keywords used to perform Google Trends searches, using the rtweet R package^22^. More specifically, we used the search_tweets function of the R package, which allows us to specify the keywords used to filter and select tweets. Three batches of 18,000 tweets were collected 3 times a day, everyday, due to the restrictions on the maximum number of tweets to be downloaded in each query by rtweet. Tweets can also be collected directly via Twitter’s standard search application programming interface (API), with the advantage that the user is not bound to any specific software, as shown by other researchers in the literature who also employ different Covid-19—related search words to filter relevant tweets^23^. For our analysis, we proceeded with a meticulous data cleansing, which lead to a considerable reduction of the final number of tweets that we included in our dataset. We carefully discarded any tweets not directly related to the Covid-19 pandemic, since, for example, the keywords *first wave*, *second wave* and *variant* led to some irrelevant tweets and noisy information. We removed tweets written from outside the UK and those produced from locations with less than 10 tweets. We also deleted any duplicate tweets and those sent by automated accounts which contained factual information about daily case numbers or retweets of news stories. Finally, we removed tweets directly addressed to foreign political leaders or politicians, obtaining a final large Twitter dataset of 577,231 tweets. From the Twitter data, we considered the total number of tweets as well as the sentiment scores calculated using two different lexicons: Bing and Afinn^24^, which are available in the R tidytext package^25^. The Bing lexicon splits words into positive or negative. The Bing sentiment score for each tweet is calculated by counting the number of positive words used in each tweet and subtracting from this the number of negative words. The Afinn lexicon scores words between $\pm \, 5$. The Afinn sentiment score is calculated by multiplying the score of each word by the number of times it appears in the tweet; these scores are then summed to derive the overall sentiment score. Therefore, from online-gathered information (b) we obtained the following 4 variables: Afinn sentiment score (Afinn), Bing sentiment score (Bing), Google trends (Google) and the total number of tweets (Tweets).

## Methodology

The copula is a function that allows us to bind together a set of marginals, to model their dependence structure and to obtain the joint multivariate distribution^26–29^. Sklar’s theorem^30^ is the most important result in copula theory. It states that, given a vector of random variables ${{\textbf {X}}}=(X_1, \ldots , X_d)$, with *d*-dimensional joint cumulative distribution function $F(x_1, \ldots ,x_d)$ and marginal cumulative distributions (cdf) $F_j(x_j)$, with $j=1, \ldots , d$, a *d*-dimensional copula *C* exists, such that

$$\begin{aligned} F(x_1, \ldots ,x_d) = C(F_1(x_1), \ldots , F_d(x_d); \varvec{\theta }), \end{aligned}$$

where $F_j(x_j) = u_j$, with $u_j \in [0,1]$ are called *u-data*, and $\varvec{\theta }$ denotes the set of parameters of the copula. The joint density function can be derived as

$$\begin{aligned} f(x_1, \ldots ,x_d) = c(F_1(x_1), \ldots , F_d(x_d); \varvec{\theta }) \cdot f_1(x_1) \cdots f_d(x_d), \end{aligned}$$

where *c* denotes the *d*-variate copula density. The copula allows us to determine the joint multivariate distribution and to describe the dependencies among the marginals, that can potentially be all different and can be modelled using distinct distributions. As it is clear from the previous equations, differently from regression models, the variables $X_1, \ldots , X_d$ hold the same role and all of them, in turn, can be used to calculate predictions.

In this paper, we adopt the 2-steps inference function for margins (IFM) approach^31^, estimating the marginals in the first step, and then the copula, given the marginals, in the second step.

### Marginal models

Given the different characteristics of the ten marginals, we fitted different models for each of the ten time series. Further, we extracted the residuals $\varepsilon _j$, with $j = 1, \ldots , d$, from each marginal model and we applied the relevant distribution functions to get the *u-data* $F_j(\varepsilon _j) = u_j$ to be plugged into the copula.

- *New admitted patients* (Admissions): The best fitting model for the Admissions marginal was the SHASHo2 model. This model belongs to the family of GAMLSS distributions, which stands for Generalised Additive Models for Location, Scale and Shape. GAMLSS are very flexible models, which include a wide range of continuous and discrete distributions. The SHASHo2 model is also known as Sinh-Arcsinh original type 2 distribution and depends on four parameters: $\mu$ the location parameter, $\sigma$ the scaling parameter, $\nu$ the skewness parameter and $\tau$ the kurtosis parameter^32^. We assumed that the parameter $\mu$ of the SHASHo2 model is related to time, as explanatory variable, through an appropriate link function, with coefficient $\beta$^33^.
- *Afinn sentiment score* (Afinn): We fitted the Afinn marginal with a reparametrized version of Skew Student *t* type 3 model (SST), which, similarly to the previous marginal, belongs to the family of GAMLSS distributions and depends on four parameters: the mode ($\mu$), scaling ($\sigma$), skewness ($\nu$) and kurtosis ($\tau$)^34^. Similarly to the SHASHo2 model, for the SST model we assumed that the parameter $\mu$ is related to time, as explanatory variable, through an appropriate link function, with coefficient $\beta$.
- *Bing sentiment score* (Bing): The best model for Bing was the Normal-Exponential-*t* (NET) distribution. This is again a four parameter continuous distribution belonging the GAMLSS family^35^. The parameters are: mean ($\mu$), scaling ($\sigma$), first kurtosis parameter ($\nu$) and second kurtosis parameter ($\tau$). As with the previous marginals, we assumed that the parameter $\mu$ of the NET model is related to time.
- *Number of positive cases* (Cases): We fitted the Cases marginal with an ARIMA-GARCH model with Student’s t innovations. This model combines the features of the autoregressive integrated moving average (ARIMA) model with the generalized autoregressive conditional heteroskedastic (GARCH) model, allowing us to capture time series volatility over time^36^. The GARCH model is typically denoted as GARCH(*p*, *q*), with parameters *p* and *q*, where *p* is the number of lag residuals errors and *q* is the number of lag variances.
- *Number of deaths* (Deaths): The best model for the Deaths marginal was the SHASHo model, whose acronym stands for Original sinh–arcsinh distribution. This model is very similar to the SHASHo2. As for the other marginals fitted with GAMLSS-type models, we assumed that the parameter $\mu$ of the SHASHo depends on time.
- *Google trends* (Google): Since Google includes values equal to zero, we fitted a Tweedie Generalised Linear Model for this marginal^37^. The Tweedie distribution has nonnegative support and can have a discrete mass at zero, making it useful to model responses that are a mixture of zeros and positive values.
- *Number of hospital cases* (Hospital): The best model for the Hospital marginal was the ARIMA-GARCH model with Student’s t innovations.
- *Number of patients on ventilation* (ICU_Beds): The best fitting model for the ICU_Beds marginal was the SHASHo model.
- *Total number of tweets* (Tweets): We fitted the Tweets marginal with a Skew Exponential Power type 4 (SEP4) model, which is a four parameter distribution belonging to the GAMLSS family^33^. Here we assumed that the parameter $\mu$ is related to time, as explanatory variable.
- *Number of tests* (VirusTests): The best model for the VirusTests marginal was the ARIMA-GARCH model with Student’s t innovations, fitted on the the number of tests adjusted by 1000.

### Vine copula model

A *vine copula* (or *vine*) represents the pattern of dependence of multivariate data via a cascade of bivariate copulas, allowing us to construct flexible high-dimensional copulas using only bivariate copulas as building blocks. The vine structure is highly flexible, since the bivariate copula densities can belong to any family^12^.

Two particular types of vines are the Gaussian vine (that we named model (ii)) and the Independence vine (model (iii)). The first one is constructed using solely Gaussian bivariate pair-copulas as building blocks, such that each conditional bivariate copula density is a Gaussian copula. The second type is the independence vine, which is constructed using only independence pair-copulas, that are simply given by the product of the marginal distributions of the random variables. In this latter case each conditional bivariate copula density is an Independence copula, implying absence of dependence between the variables.

In order to estimate the vine, its structure as well as the copula parameters have to be specified. A sequential approach is generally adopted to select a suitable vine decomposition, specifying the first tree and then proceeding similarly for the following trees. For selecting the structure of each tree, we followed the approach based on the maximal spanning tree algorithm^38,39^. Given the selected tree structure, a copula family for each pair of variables is identified using the Akaike Information Criterion (AIC), or the Bayesian Information Criterion (BIC). This choice is typically made amongst a large set of families, comprising elliptical copulas (Gaussian and Student’s t) as well as Archimedean copulas (Clayton, Gumbel, Frank and Joe), their mixtures (BB1, BB6, BB7 and BB8) and their rotated versions, to cover a large range of possible dependence structures^26,27^. The copula parameters $\varvec{\theta }$ for each pair-copula in the vine are estimated using the maximum likelihood (MLE) method^38^. The vine estimation procedure is repeated for all the trees, until the vine is completely specified.

### Out-of-sample predictions

In order to evaluate the suitability of the proposed vine copula model in relation to other methods, we produced 1-day-ahead out-of-sample predictions and we compared them to the original data. Let ${{\textbf {X}}} = \{ {{\textbf {X}}}_t; t = 1,.., T \}$ be the 10-dimensional time series of Covid-19 and social media data. Our aim is to forecast ${{\textbf {X}}}_{T+1}$ based on the information available at time *T*^40^. Before fitting the vine, we extracted the residuals from the marginals and obtained the *u-data*. Next, after fitting the vine, we simulated *M* realizations from the vine copula. Hence, we calculated the predicted values for each simulation, using the inverse cdf and the relevant fitted marginal models. More precisely, we applied the inverse transformation to the *M* realizations from the vine copula to obtain the residuals which we then plugged into the marginal models to get the predicted values of the official variables. Then, we calculated the average prediction for all simulations $\hat{{{\textbf {X}}}}_{T+1}^{Avg}$ and use it to forecast ${{\textbf {X}}}_{T+1}$. The prediction interval of level $(1 - \alpha ) \in (0, 1)$ for ${{\textbf {X}}}_{T+1}$ was calculated by taking the estimated quantiles of order $\alpha /2$ and $1 - \alpha /2$ amongst the simulated data. We denote by $\hat{{{\textbf {X}}}}_{T+1}^l$ and $\hat{{{\textbf {X}}}}_{T+1}^u$ the lower and upper values of the prediction intervals.

In order to compare and contrast the accuracy of predictions for different models, we made use of two indicators: the mean squared error (MSE) to evaluate point forecasts and the mean interval score (MIS)^41^, to assess the accuracy of the prediction intervals. The MSE for each variable $j = 1,\ldots , d$ was calculated as follows

$$\begin{aligned} \text{ MSE}_j = \frac{1}{S} \sum _{t=T+1}^{T+S} (x_{t,j} - {\hat{x}}_{t,j})^2, \end{aligned}$$

where $x_{t,j}$ is the observed value for each variable at each time point *t*, ${\hat{x}}_{t,j}$ is the corresponding predicted value, $T+1$ denotes the first predicted date, while $T+S$ indicates the last predicted date. The 95% MIS for each variable, at level $\alpha =0.05$, was computed as

$$\begin{aligned} \text{ MIS}_j = \frac{1}{S} \sum _{t=T+1}^{T+S} \left[ ({\hat{x}}_{t,j}^u - {\hat{x}}_{t,j}^l) + \frac{2}{\alpha } ({\hat{x}}_{t,j}^l - x_{t,j}) \mathbbm {1}(x_{t,j} < {\hat{x}}_{t,j}^l) + \frac{2}{\alpha } (x_{t,j} - {\hat{x}}_{t,j}^u) \mathbbm {1}(x_{t,j} > {\hat{x}}_{t,j}^u) \right] , \end{aligned}$$

where ${\hat{x}}_{t,j}^l$ and ${\hat{x}}_{t,j}^u$ denote, respectively, the lower and upper limits of the prediction intervals for each variable at each time point, and $\mathbbm {1}(\cdot )$ is the indicator function.

## Result analysis and discussion

We now present the results of the analysis of the official and online-retrieved Covid-19 data.

### Twitter wordclouds

First, as an exploratory tool, we analysed the information gathered on Twitter, cleaning and stemming the tweets and producing graphical representations of the data using wordclouds. We point out that the analysis of wordclouds does not contribute to prediction, but it is a useful step to provide insights on the online-retrieved text information.

**Figure 1:**
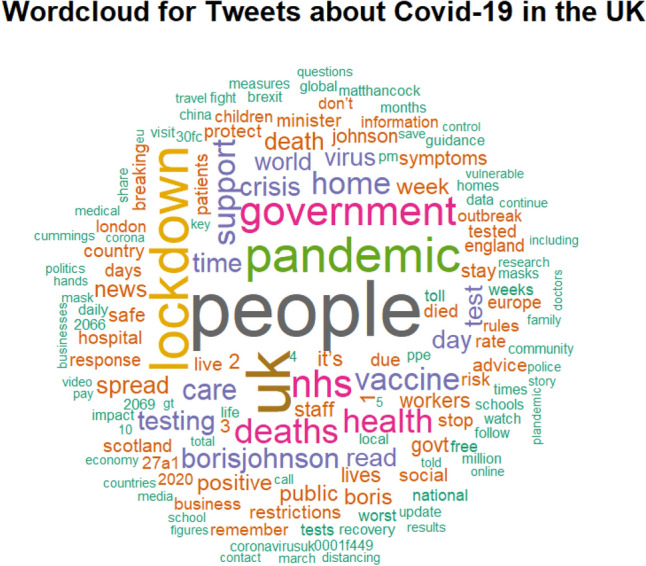
Wordcloud of the UK Covid-19 data. Figure created by the authors using the package wordcloud of the R software version 4.2.2. https://cran.r-project.org/.

Figure 1 displays the wordcloud obtained from the collected tweets discussing Covid-19 in the UK. The most frequent words are related to “people” and the effects of the pandemic on them. We can also notice the names of the most prominent politicians and words related to political decisions.

**Figure 2:**
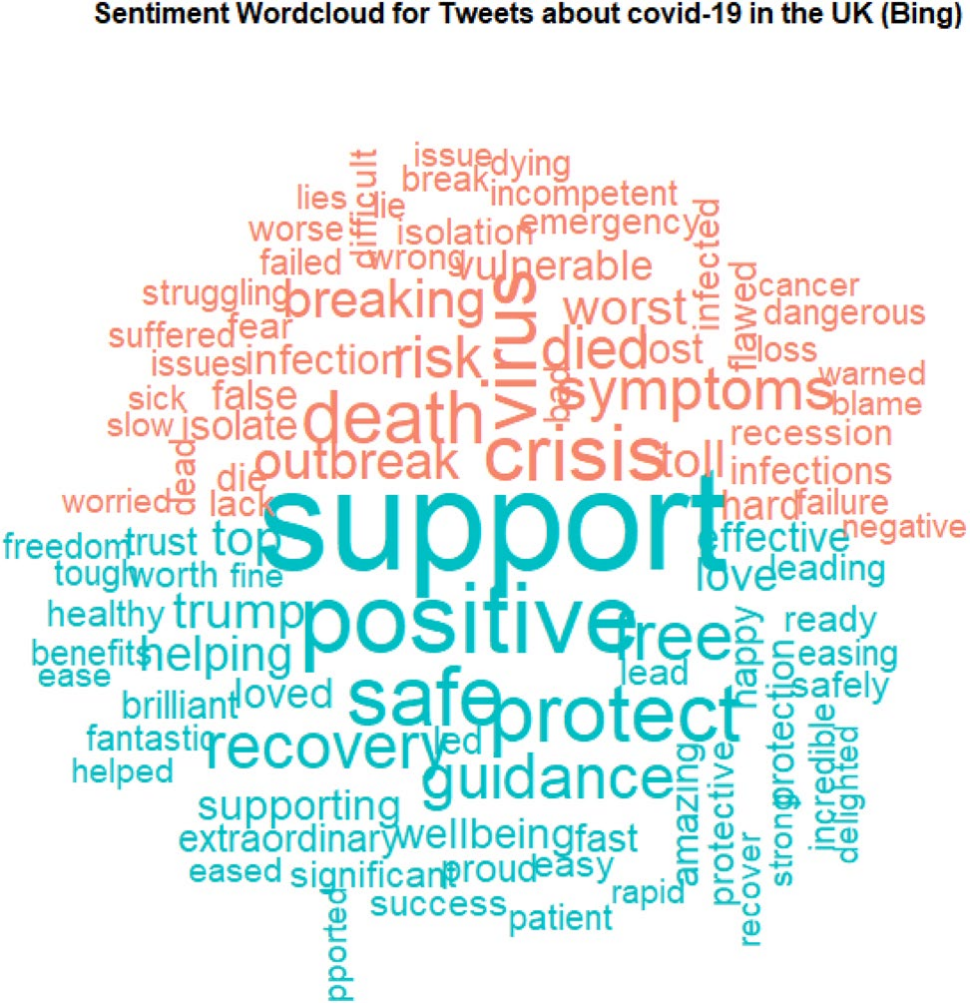
Sentiment wordcloud for the UK Covid-19 data obtained with the Bing method. Positive words are in blue, negative words are in pink. Figure created by the authors using the package wordcloud of the R software version 4.2.2. https://cran.r-project.org/.

Figure 2 shows the sentiment wordcloud created from the collected tweets, obtained with the Bing method. This data visualization highlights the positive words in blue and the negative words in pink. The most popular positive words are related to the “support” received throughout the pandemic, while the most popular negative words are related to the worst consequences of Covid-19 on the health of individuals.

### Marginals estimation

**Figure 3:**
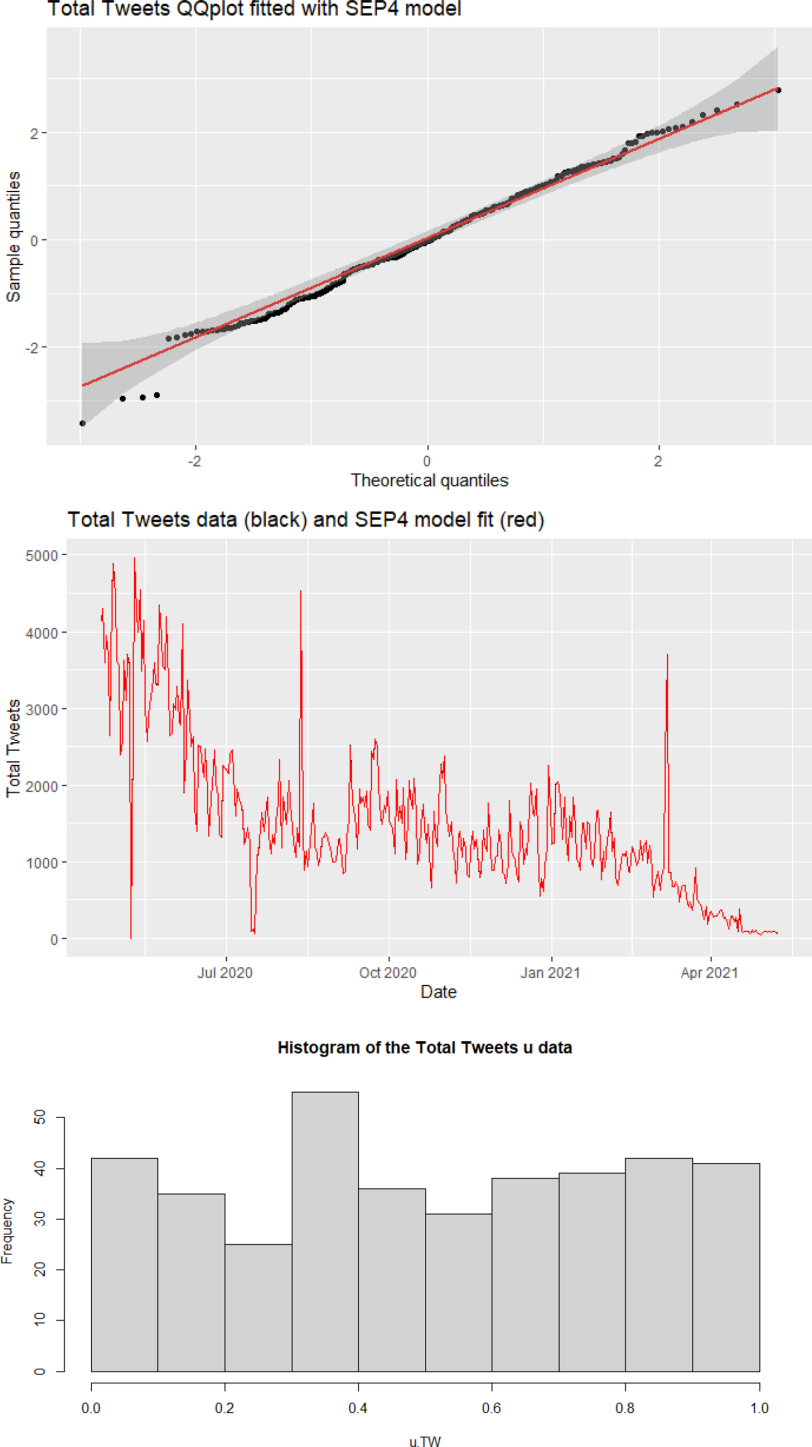
Plot illustrating the fit of the residuals for the Tweets marginal. Top plot: QQ-plot comparing the Gaussian theoretical quantiles with sample quantiles. Middle plot: observed time series (black line) and in-sample predictions obtained form the fitted SEP4 model (red line). Bottom plot: Histogram of the resulting *u-data*.

As an example, Fig. 3 shows the fit of the residuals for the Tweets marginal. The top panel displays the QQ-plot comparing the Gaussian theoretical quantiles with the sample quantiles, the middle panel illustrates the observations (black line) and in-sample predictions obtained from the fitted SEP4 model (red line), while the bottom panel shows the histogram of the resulting *u-data*. The plots clearly show an excellent fit of the SEP4 model to the marginal, as demonstrated by the points in the QQ-plot aligning well to the main diagonal, the in-sample predictions overlapping the observed data and the shape of the *u-data* histogram being close to a uniform pattern.

### Vine estimation

Once the marginals were estimated, we derived the corresponding *u-data* from the residuals. Then, we carried out fitting and model selection for the vine copula.

**Figure 4:**
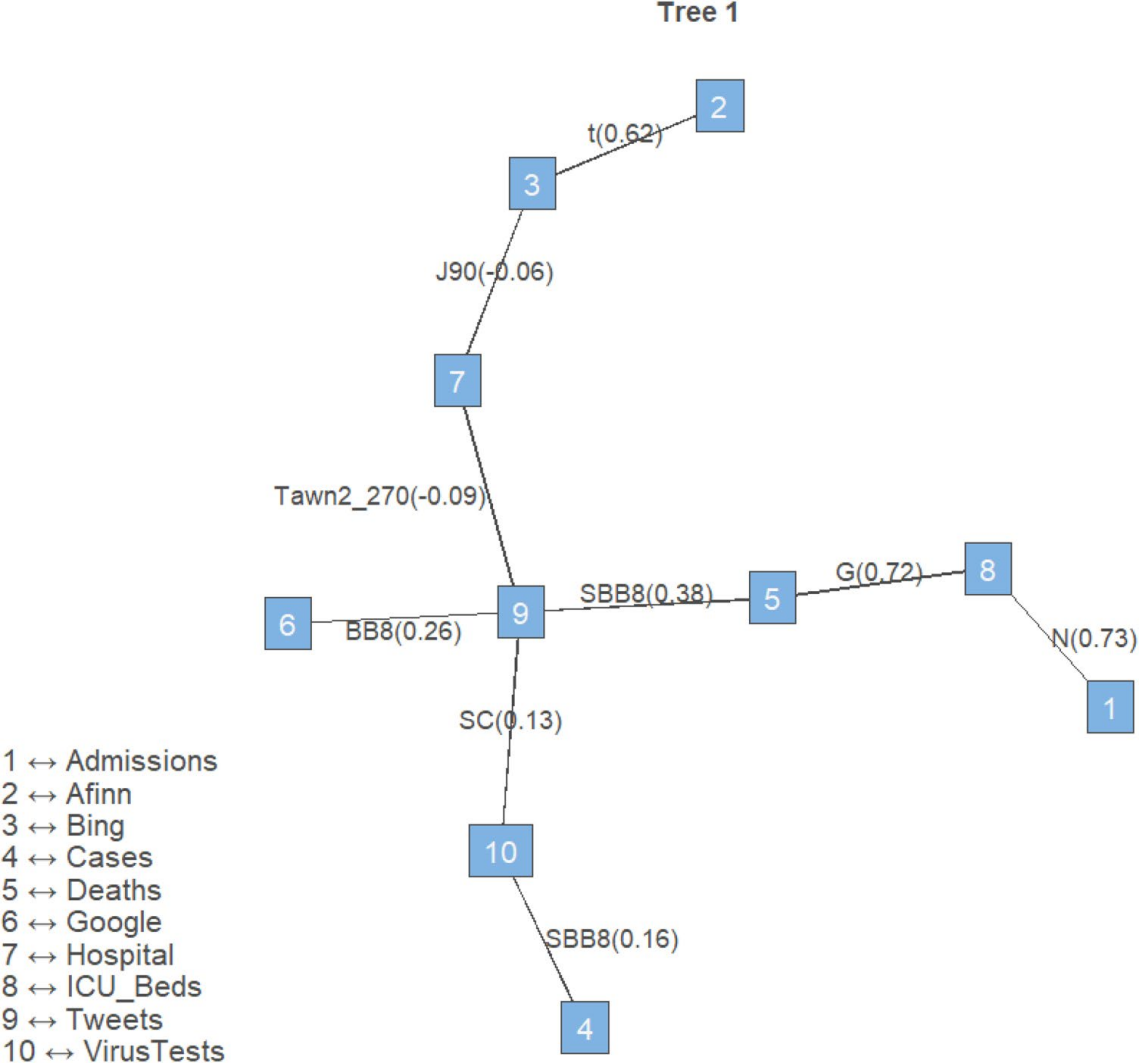
First tree of the vine copula for the Covid-19 data. The legend shows the names of the variables displayed on each node. The pair-copula families are shown on the edges and the Kendall’s $\tau$s are given in brackets.

Figure 4 displays the first tree of the vine copula for the Covid-19 data, that we previously named model (i). The nodes are denoted with blue squares, with the numbers corresponding to the margins reported on them. On each edge, the plot shows the name of the selected pair copula family and the estimated copula parameter expressed as Kendall’s $\tau$. In order to estimate the vines, we adopted the Kendall’s $\tau$ criterion for tree selection, the AIC for the copula families selection and the MLE method for estimating the pair copula parameters. As it is clear from Fig. 4, the total number of tweets plays a central role in the vine, linking official Covid-19 to social media variables. The total number of tweets and Google searches are contiguously related. Likewise, the sentiment scores Bing and Afinn are directly associated. Tweets is also directly connected to the number of deaths, the total number of tests and the number of hospital cases. The symmetric Gaussian copula, which is often employed in traditional multivariate modelling, was only identified once as the best fitting copula, to link the number of patients on ventilation and the number of new admissions. This suggests that model (i), with different pair copula families, better fits the data compared to model (ii), which assumes all Gaussian pair copula families. On the contrary, the selected copula families include the Student’s t copula, which is able to model strong tail dependence, the Tawn copula and Archimedean copulas such as the Clayton, Gumbel and Joe, that are able to capture asymmetric dependence, and mixture copulas such as the BB8 (Joe–Frank), that can accommodate various dependence shapes. Most of the associations between the variables are positive. The strongest associations are between the official Covid-19 variables ICU_Beds and Admissions, between ICU_Beds and Deaths and between the Bing and Afinn sentiment scores. Also, Deaths and Tweets are mildly associated. The results suggest that associations between official (a) and social media data (b) are present and that the independence model (iii), which assumes no relationship between different sources of information is not appropriate for the data.

### Out-of-sample prediction results

In this Section we constructed out-of-sample predictions using the proposed vine methodology (model (i)), which integrates official and social media Covid-19 variables. We then compared the predictions obtained with our methodology with those yielded using two traditional approaches. The former is based on vines built exclusively using Gaussian pair copulas (model (ii)), which are the most common in applications, but are restricted to dependence symmetry and absence of tail dependence. The latter approach assumes independence among the ten time series under consideration (model (iii)) and therefore calculates predictions ignoring any association between official (a) and online information (b). Since the vine approach allows all the variables to hold the same role, we calculated predictions for each variable in turn based on the remaining variables.

Out-of-sample predictions based on the proposed model were constructed considering the vine copula estimated until the ${1}{{\text{st}}}$ April 2021 and using it to predict the period between the ${2}{{\text{nd}}}$ April 2021 and the ${9}{{\text{th}}}$ May 2021.

Tables 1 and 2 list the MSE and MIS values calculated for each variable. The second columns show the vine copula (model (i)) results, the third columns show the results assuming all Gaussian pair-copulas (model (ii)) and the fourth columns show the results assuming independence among variables (model (iii)). The MSEs and MISs of the best performing approaches for each variable are highlighted in boldface. Tables 1 and 2 show a similar model performance across the ten variables. According to both the MSE and MIS indicators, the vine copula approach (i) outperforms the other two approaches (ii) and (iii) for predicting the official (a) variables Deaths, Hospital and VirusTest. The Gaussian vine approach (ii) also performs well with several variables, while the independent approach (iii) seems to exceed the other two approaches only with the variables Admissions and Afinn.

**Table 1:** MSEs calculated for each variable.

Marginal	Vine copula	Gaussian	Independent
Admissions	12,510.57	12,515.63	**12,506.15**
Afinn	1.0226	1.0667	**0.9766**
Bing	**0.2417**	0.2597	0.2452
Cases	577,363	**577,332.3**	577,401.1
Deaths	**886.7999**	890.7506	887.5182
Google	385.3506	**382.9412**	384.9532
Hospital	**1348411**	1,348,573	1,348,496
ICU_Beds	49,150.17	**49,142.98**	49,144.45
Tweets	17,593.4	**17,583.2**	17,585.62
VirusTests	**292,876**	292,943.3	292,902.5

The figures show the vine copula results (second column), the results assuming all Gaussian pair-copulas (third column), and assuming independence among variables (fourth column).

The MSEs of the best performing approaches for each variable are in boldface.

**Table 2:** MISs calculated for each variable.

Marginal	Vine copula	Gaussian	Independent
Admissions	25.5155	25.5208	**25.5101**
Afinn	0.2510	0.2587	**0.2461**
Bing	0.1187	0.1231	**0.1148**
Cases	173.1329	**173.1258**	173.1414
Deaths	**6.5837**	6.5991	6.5867
Google	4.5348	**4.1436**	4.1482
Hospital	**257.4757**	257.4917	257.4841
ICU_Beds	49.3001	**49.2965**	49.2972
Tweets	28.9344	**28.9253**	28.9278
VirusTests	**120.5653**	120.5799	120.5709

The figures show the vine copula results (second column), the results assuming all Gaussian pair-copulas (third column), and assuming independence among variables (fourth column).

The MISs of the best performing approaches for each variable are in boldface.

The 6 official (a) Covid-19 variables considered in this paper, Admissions, Cases, Deaths, Hospital, ICU_Beds and VirusTests, are generally better predicted by the vine method (i), as opposed to the Gaussian (ii) and independence (iii) methods. This last approach assumes no dependence between any of the variables involved in the model. Hence, this approach indicates the absence of any association between the official (a) and the social media (b) variables, implying the lack of contribution of online-generated information in predicting the official Covid-19 variables. On the contrary, the vine approach assumes the presence of a dependence structure between the variables and, in particular, between the official (a) and social media (b) insights. In particular, the highly flexible vine model (i) allows us to model asymmetric and tail dependence; while the Gaussian vine model (ii) only allows for symmetric and no tail dependence. Therefore, the better performance of the vine (i) and (ii) compared to the independence model (iii) demonstrates the usefulness of social media information in forecasting official Covid-19 variables.

The prediction of the 4 online-generated information (b) considered in this paper (Afinn, Bing, Google and Tweets) also benefits from data integration. Indeed, most of the social media variables are more accurately forecasted by the vine model, particularly the Gaussian one (model (ii)). This indicates that the Gaussian approach, characterized by a symmetric dependence structure, is flexible enough to model the social media variables.

## Concluding remarks

In this paper, we propose a new methodology aimed at obtaining more accurate forecasts compared to traditional approaches, for variables measuring the Covid-19 dynamics. The proposed methodology is based on the integration of Covid-19 variables collected from official UK sources with online generated social media insights, relevant to the same geographical area.

The results show that the vine copula method generally outperforms two other traditional approaches (more precisely, the Gaussian vine copula approach and the independence approach) for predicting the official pandemic variables indicating the number of deaths, hospital admissions and tests, demonstrating that the proposed methodology is able to leverage social media information to obtain accurate predictions of Covid-19 effects.

## Data Availability

The datasets analysed during the current study are available in the GOV.UK repository, https://coronavirus.data.gov.uk, in the Johns Hopkins University repository, https://coronavirus.jhu.edu/region/united-kingdom, via the Twitter API, https://developer.twitter.com, and via the Google Trends API, https://trends.google.com.

## Code availability

The code that implements the methodology described in the paper is available in the GitHub repository https://github.com/laurenansell/A-New-Data-Integration-Framework-for-Covid-19-Social-Media-Information.
